# Adsorption of Inositol Phosphate on Hydroxyapatite Powder with High Specific Surface Area

**DOI:** 10.3390/ma15062176

**Published:** 2022-03-15

**Authors:** Hirogo Minamisawa, Yoshiyuki Kojima, Mamoru Aizawa

**Affiliations:** 1 Organization for the Strategic Coordination of Research and Intellectual Properties, Meiji University, 1-1-1 Higashimita, Tama-ku, Kawasaki, Kanagawa 214-8571, Japan; h_minamisawa@meiji.ac.jp; 2 Department of Materials and Applied Chemistry, Faculty of Science and Engineering, Nihon University, 1-8, Kanda-Surugadai, Chiyoda-ku, Tokyo 101-8308, Japan; kojima.yoshiyuki@nihon-u.ac.jp; 3 Department of Applied Chemistry, School of Science and Technology, Meiji University, 1-1-1 Higashimita, Tama-ku, Kawasaki, Kanagawa 214-8571, Japan

**Keywords:** Hydroxyapatite, ultrasonic irradiation, inositol phosphate, chelate-setting cement, adsorption

## Abstract

Chelate-setting calcium-phosphate cements (CPCs) have been developed using inositol phosphate (IP6) as a chelating agent. However, the compressive strength of the CPC fabricated from a commercially available hydroxyapatite (HAp) powder was approximately 10 MPa. In this study, we miniaturized HAp particles as a starting powder to improve the compressive strength of chelate-setting CPCs and examined the adsorption properties of IP6 onto HAp powders. An HAp powder with a specific surface area (SSA) higher than 200 m^2^/g (HApHS) was obtained by ultrasonic irradiation for 1 min in a wet synthesis process, greatly improving the SSA (214 m^2^/g) of the commercial powder without ultrasonic irradiation. The HApHS powder was found to be a B-type carbonate-containing HAp in which the phosphate groups in apatite were replaced by carbonate groups. Owing to the high SSA, the HApHS powder also showed high IP6 adsorption capacity. The adsorption phenomena of IP6 to our HApHS and commercially available Hap powders were found to follow the Freundlich and Langmuir models, respectively. We found that IP6 adsorbs on the HApHS powder by both physisorption and chemisorption. The fine HapHS powder with a high SSA is a novel raw powder material, expected to improve the compressive strength of chelate-setting CPCs.

## 1. Introduction

Hydroxyapatite (Ca_10_(PO_4_)_6_(OH)_2_; HAp), the main component of human bones and teeth, is known as a bioceramic and is commonly used as an artificial bone and tooth root [1,2]. Artificial bones put to practical use in the medical field have various forms and shapes, such as granules [3], blocks [4], cylinders [5], porous bodies [6], and paste (cement) [7,8,9], and suitable ones are selected depending on the application.

Among various forms, cements are set over time from a soft paste, which has the advantage that it can be molded to fit the shape of the bone defect. In addition, the use of cement has attracted considerable attention because it can be supplemented by injectors, allowing for minimally-invasive surgery [7,8,9]. In general, calcium-phosphate cement (CPC) for medical applications is prepared by mixing calcium phosphate powder with an appropriate solution [10,11,12,13]. Brown and Chow reported a cement fabricated using acidic calcium hydrogen phosphate (CaHPO_4_) and basic tetracalcium phosphate (Ca_4_O(PO_4_)_2_) as starting cement powders, and the chemical reaction that occurs during mixing these with a solution produces HAp, which sets the cement [14]. However, this setting reaction may cause inflammation around the tissue due to the acid-base reaction [15].

One of the authors, Aizawa and his colleagues, have reported the fabrication of a novel CPC cement using inositol phosphate (C_6_H_6_(OPO_3_H_2_)_6_; IP6) as a chelating agent [16,17,18]. IP6 is abundant in grains such as wheat, rice, and corn, and it is commonly used as a food additive [19,20]. Therefore, it is safe for use in vivo. Furthermore, the phosphate group in IP6 is known to chelate against Ca^2+^ ions in HAp. This chelating ability is as strong as that of ethylenediaminetetraacetic acid (EDTA) [18,21]. The chelate-setting cement is mixed with the HAp particles with IP6 surface modification and pure water, and it sets due to the chelating effect of IP6. In this manner, the cement was prepared without an acid-base reaction, and the problems of conventional cement fabrication were solved. However, in our previous study [16], the compressive strength of cements with IP6 surface modification on HAp powders with various specific surface areas (SSA) was low, ranging from approximately 4 to 10 MPa. This was approximately one-fifth of the compressive strength of commercial cements. Compressive strength could be enhanced by increasing the SSA [17].

Kojima et al. reported the synthesis of HAp with nanometer-order particle sizes and high SSA [22]. The HAp powder with an SSA of approximately 300 m^2^/g was synthesized by ultrasonic irradiation of a solution of calcium hydroxide (Ca(OH)_2_) suspension and phosphoric acid (H_3_PO_4_). Moreover, the high SSA of the HAp powder improved its functionality as a catalyst [23].

We used this HAp powder with a high SSA as a starting material for chelate-setting cement and to improve the compressive strength. In general, one can imagine two causes leading to an improved compressive strength of the cement: (1) the high SSA increases the amount of IP6 adsorbed (increasing the number of IP6 binding sites among particles due to miniaturization of the HAp particles), and (2) the nanoparticles increase the particle filling (improving the particle filling in hardened cement by miniaturization of the same particles).

In this study, as a first step to developing high-strength chelate-setting CPCs based on HAp, we optimized the conditions for the synthesis of HAp powder with a high specific surface area (HApHS), In addition, the amounts of IP6 adsorbed on the resulting HApHS versus commercially available HAp powders was compared with respect to the adsorption mechanism.

## 2. Materials and Methods

### 2.1. Synthesis of Hydroxyapatite Powder with High Specific Surface Area

The HApHS powder was synthesized using Ca(OH)_2_ and H_3_PO_4_ in an aqueous solution by a wet process [22].

10Ca(OH)_2_ + 6H_3_PO_4_ → Ca_10_(PO_4_)_6_(OH)_2_ + 18H_2_O

Ca(OH)_2_ powder (FUJIFILM Wako Pure Chemical Co., Osaka, Japan) was added to water (200 cm^3^) to prepare a suspension. The suspension was sonicated for 0, 1, 2, 3, 4, and 5 min using an ultrasonic homogenizer UD-200 (TOMY SEIKO CO., Ltd., Tokyo, Japan). The horn attached to the homogenizer was a Micro Tip TP-040 (TOMY SEIKO CO., Ltd., Tokyo, Japan), with a diameter of 3.2 mm and an output power of 20 W. The H_3_PO_4_ solution (200 cm^3^, 0.1 mol/dm^3^) was prepared by diluting the H_3_PO_4_ reagent (FUJIFILM Wako Pure Chemical Co., Osaka, Japan) with pure water. After adding the H_3_PO_4_ solution to the ultrasonically irradiated Ca(OH)_2_ suspension to obtain a Ca/P molar ratio of 1.67, the reaction was carried out by stirring for 1 h. The mixture was then centrifuged (9000 rpm) for 5 min and the supernatant liquid was removed. The HApHS(X) powders (X: 0, 1, 2, 3, 4, and 5 min) were obtained by freezing the precipitate overnight and then freeze-drying it for one day.

### 2.2. HApHS Powder Characterization

The median diameter of the sonicated Ca(OH)_2_ powder was measured using a laser particle size analyzer (LA-300, HORIBA, Ltd., Kyoto, Japan). The crystalline phase of the synthesized powder was identified using an X-ray diffractometer (XRD; MiniFlex, Rigaku, Tokyo, Japan) equipped with a Cu-K_α_ radiation source (λ = 0.151418 nm) operated at 30 kV and 15 mA. The XRD patterns were collected in the range of 2*θ* = 10–60° with a step size of 0.04° and a counting time of 4 s/step. The crystalline phase was identified by comparison with the ICDD reference patterns for HAp (#09-0432).

The functional groups of the synthesized powder were assigned using a Fourier transform infrared (FT-IR) spectrophotometer (IR Prestige-21, Shimadzu Co., Kyoto, Japan) in the wavenumber range of 400–4000 cm^−1^ with a resolution of 4 cm^−1^ using potassium bromide pellets to disperse the sample.

The Ca/P atomic ratio was calculated after each element was determined by inductively coupled plasma atomic emission spectroscopy (ICP-AES; SPS7800, SII Nano Technology Co., Chiba, Japan). The powder was dissolved in aqueous nitric acid before the ICP-AES measurement.

The SSA was determined using a dynamic sorption surface area analyzer (FlowSorb III, Micromeritics Instrument Co., GA, USA). For this measurement, the Brunauer–Emmett–Teller (BET) method with nitrogen gas was used [24].

The particle morphology was observed using transmission electron microscopy (TEM; JEM-2100F, JEOL Ltd., Tokyo, Japan) at an accelerating voltage of 200 kV. The samples for TEM observation were prepared by dispersing the powders in ethanol and collecting them on copper grids that have been reinforced with carbon (400 mesh, JEOL Ltd., Tokyo, Japan).

### 2.3. Surface Modification of HApHS Powder with IP6

The shaking time of the synthesized powder needed to modify the surface of IP6 was found using the following procedure. The HApHS powder and a 1000 ppm IP6 solution (10 cm^3^) were added to a centrifuge tube (15 cm^3^). For a 1000 ppm IP6 solution, a 50% phytic acid solution (FUJIFILM Wako Pure Chemical Co., Osaka, Japan) was diluted with water and adjusted to pH 7.3 by the addition of aqueous NaOH solution [16,25]. The centrifuge tube was shaken for 0, 0.5, 1, 3, 5, 7, and 9 h at a rate of 100 times/min, holding the temperature of the water at 37 °C.

The HAp powder was then separated by centrifugation (5 min at 9000 rpm), and the supernatant was collected. The collected supernatant solution was diluted with pure water and the phosphorus was quantified by ICP-AES to determine the amount of IP6 adsorption. As a control, commercially available HAp powder (HAp-100; Taihei Chemical Industrial Co., Ltd., Osaka, Japan; hereafter referred to as “HAp powder”) with an SSA of 63 m^2^/g was used in the same experiment to evaluate the IP6 adsorption capacity. The IP6 adsorption capacities at various sampling times and at equilibrium conditions were calculated using the following equations:

(1) $$q_{t}=\frac{C_{0}-C_{t}}{m}\times V$$

(2) $$q_{e}=\frac{C_{0}-C_{e}}{m}\times V$$

where *q_t_* and *q_e_* (mg/g) are the IP6 adsorption capacities at the sampling time *t* and at equilibrium, respectively; *C*_0_ (mg/cm^3^) is the IP6 initial concentration; *C_t_* and *C_e_* (mg/cm^3^) are the IP6 concentrations at sampling time *t* and at equilibrium, respectively; *V* (cm^3^) is the volume of solution; and *m* (g) is the mass of sample powder.

### 2.4. Adsorption Isotherm of IP6

The initial concentration of the IP6 solution was varied to obtain the adsorption isotherm of IP6 [16,26]. The HApHS and HAp powders (0.3 g) were added in the IP6 solution at pH 7.3 (10 cm^3^) with initial concentration of 0, 100, 250, 500, 750, 1000, 1500, 2000, 2500, 3000, 4000, 5000, 7500, and 10,000 ppm. The solutions were then shaken at a rate of 100 times/min at 37 °C. The solutions were then centrifuged, and the precipitates were frozen overnight and freeze-dried for 24 h. Finally, the IP6/HApHS (0–10,000) powders were obtained, together with the IP6/HAp (0–10,000). Each HAp powder with IP6 modification on the surface underwent XRD analysis to identify the crystalline phase. The amount of P for the obtained supernatant was quantified by ICP-AES, and the amount of IP6 adsorbed on each HAp powder was calculated.

## 3. Results and Discussion

### 3.1. Synthesis of HApHS Powders

After ultrasonic irradiation of the Ca(OH)_2_ suspension during the wet synthesis of the HApHS powder, the particle size distribution of the suspension was measured. The SSA of the Ca(OH)_2_ powder used in this study was 12.4 m^2^/g, and its particle size was 219 nm, calculated from this SSA value on the basis of the following formula:

(3) $$S_{w}=\frac{6000}{\rho D_{m}}$$

where *S_w_* (m^2^/g) is the specific surface area; *ρ* (g/cm^3^) is density of the sample; *D_m_* (nm) is the average particle diameter. This formula was used to calculate the average particle size, assuming that the sample is a sphere [27].

**Figure 1:**
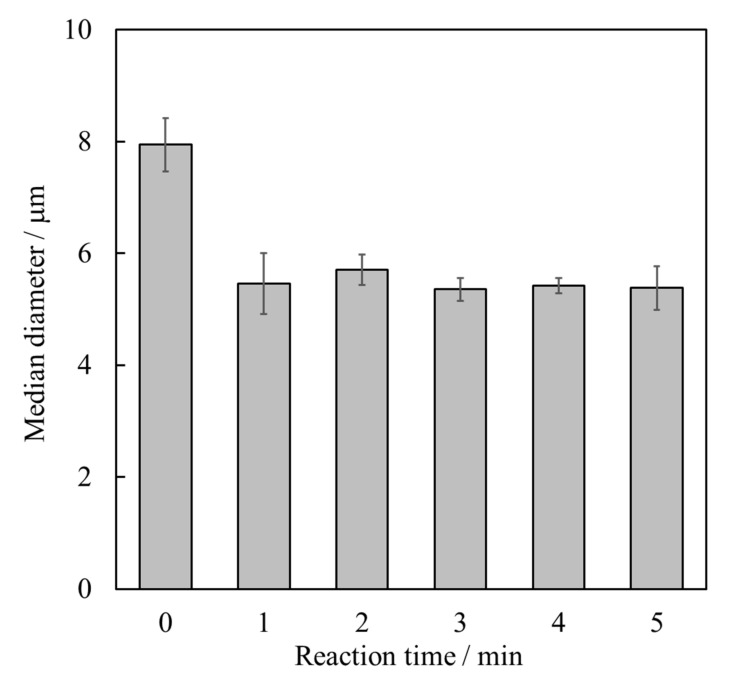
Median diameters of Ca(OH)_2_ powder after ultrasonic irradiation for different durations (error bars: ± S.D., *n* = 3).

Figure 1 shows the median particle size of the resulting HApHS powders. The results showed that the median diameter of the sample was initially 7.9 μm (0 min), decreasing to 5.5 μm after 1 min of sonication treatment (ultrasonic irradiation). The median diameter of the sample after 2 min remained. This value was significantly different from the median diameter at 0 min (Figure 1), suggesting that the ultrasonic irradiation caused a reduction in the secondary particle size of the sample and that these values of the median diameter of the calcium hydroxide powder refer to aggregated species.

**Figure 2:**
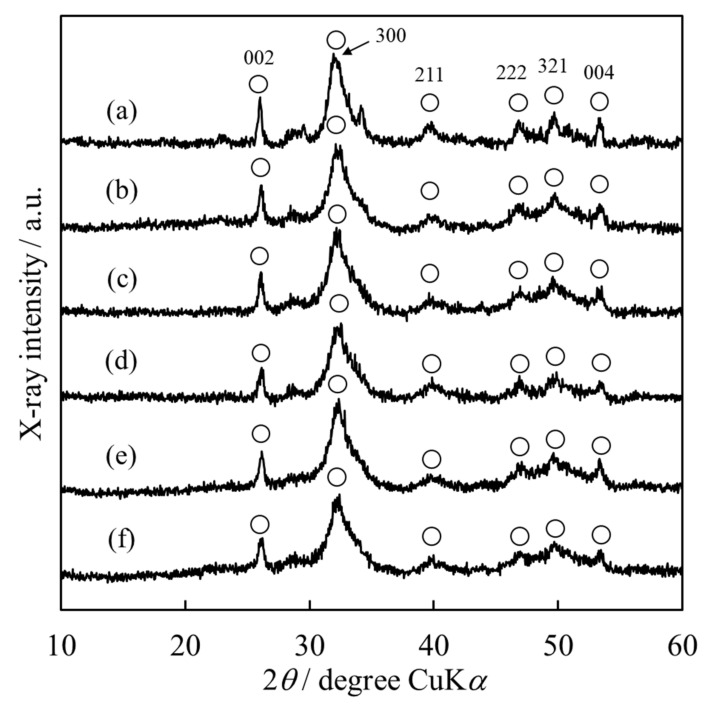
X-ray diffraction patterns of HApHS(X) powder after ultrasonic irradiation. (X: 0–5 min), (**a**) HApHS(0), (**b**) HApHS(1), (**c**) HApHS(2), (**d**) HApHS(3), (**e**) HApHS(4), (**f**) HApHS(5). 〇: HAp.

To identify the crystalline phase of the synthesized powder, we performed XRD analysis. Figure 2 shows the XRD patterns of the HApHS powders. All the powders synthesized under the conditions described seemed to be single-phase HAp. Furthermore, the broadening of each diffraction peak suggests that the powder had low crystallinity [28,29].

**Figure 3:**
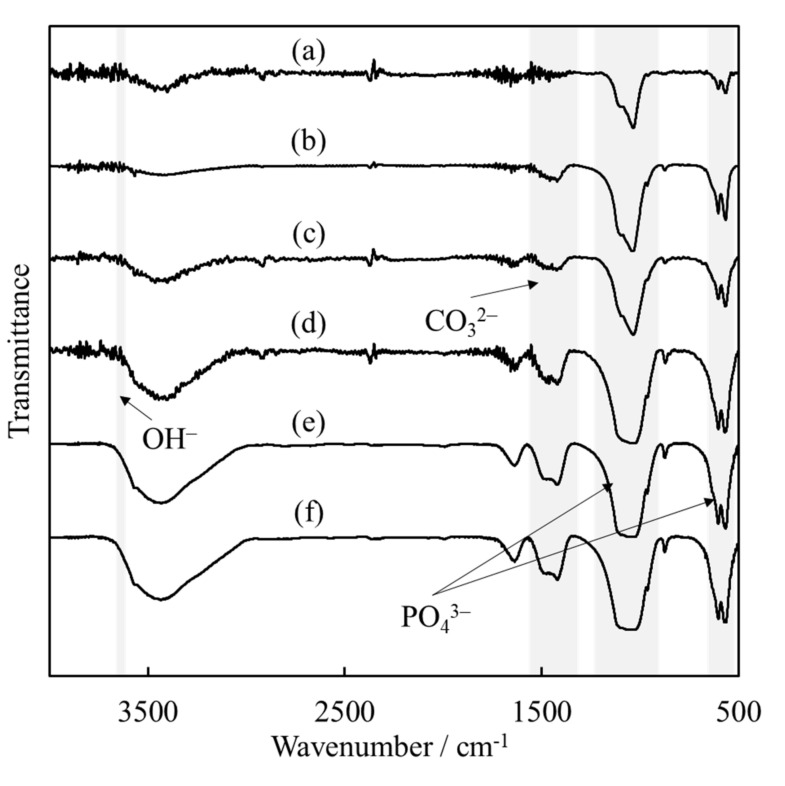
Fourier-transform infrared spectra of HApHS(X) powder after ultrasonic irradiation. (X: 0–5 min), (**a**) HApHS(0), (**b**) HApHS(1), (**c**) HApHS(2), (**d**) HApHS(3), (**e**) HApHS(4), (**f**) HApHS(5).

The assignment of functional groups was carried out by FT-IR spectroscopy. As shown in Figure 3, the absorption bands observed in all samples were assigned to OH^−^ and PO_4_^3−^ groups, which are typical signals for HAp [30]. In addition, the sonicated powders (b)–(f) show absorption peaks attributed to CO_3_^2−^ groups. These absorption peaks can be used to identify the substitution position of CO_3_^2−^ ions in apatite by their wavenumber. In more detail, carbonate-containing HAp (hereafter, CO_3_HAp) substituted with OH^−^ and PO_4_^3−^ groups are named “A-type” and “B-type”, respectively [31]. Figure 3 shows that the ultrasonic irradiated powder was a B-type CO_3_HAp, which is a typical result of wet processing. Consequently, we inferred that PO_4_^3−^ in HAp was replaced by CO_3_^2−^.

Table 1 lists the SSA and Ca/P atomic ratio values of the powders synthesized after ultrasonic irradiation for different durations. The expected theoretical Ca/P atomic ratio of HAp is 1.67 according to literature data [30]. The Ca/P atomic ratio of our sonicated powder was approximately 1.70, higher than the theoretical value, which seems caused by the replacement of PO_4_^3−^ with CO_3_^2−^.

The SSA of the HApHS(0) powder was 147 m^2^/g and was increased to 214 m^2^/g after 1 min of ultrasonic irradiation. The SSA of the synthesized powder did not vary significantly after 5 min of ultrasonic irradiation, confirming the beneficial effect of the treatment performed.

**Figure 4:**
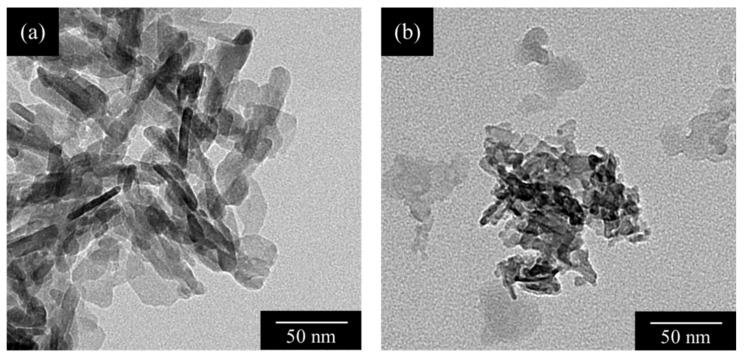
TEM images of (**a**) HApHS(0) and (**b**) HApHS(1) powders.

Figure 4 shows the TEM images of the HApHS(0) and HApHS(1) powders. The HApHS(0) powder was rod-shaped with a long diameter of the particles of approximately 20–45 nm, whereas the HApHS(1) powder did not have a characteristic shape with the long diameter being smaller than 10 nm. The SSA values of each powder in Table 1 were used to calculate the respective particle size (BET diameter) of obtaining 12.9 nm and 8.9 nm, respectively. The long diameter of HApHS(1) was in agreement with the value observed by TEM, again confirming that ultrasonic irradiation promotes the miniaturization of synthesized powder. Therefore, the SSA of HApHS(1–5) did not change; thus, we decided to use the HApHS(1) powder for the evaluation in the following study.

### 3.2. Adsorption of IP6 for Determination of Shaking Time

**Figure 5:**
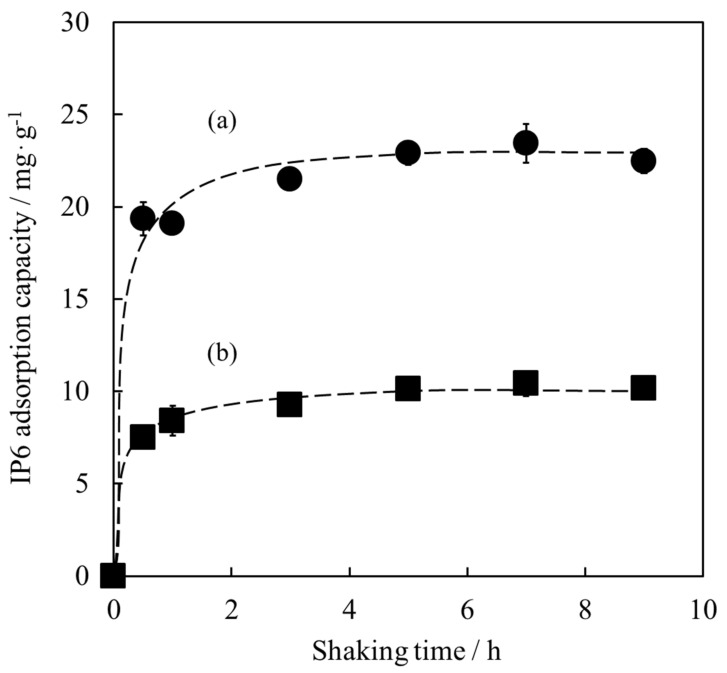
Optimization of shaking time for IP6 adsorption (error bars: ± S.D., *n* = 3) by (**a**) HApHS powder (●) and (**b**) HAp powder (■).

The HApHS(1) powder with an SSA of 208 m^2^/g was used for IP6 adsorption experiments. The SSA of the HAp powder used as control was 63 m^2^/g. First, the shaking time for the adsorption test was examined. As shown in Figure 5, both powders rapidly adsorbed IP6 within 1 h, retaining similar IP6 adsorption capacity after 5 h. The IP6 adsorption capacities of the HApHS (a) and HAp-100 (b) powders, both stirred for 5 h, were 22.9 mg/g and 10.1 mg/g, respectively.

When the IP6 adsorption reached the equilibrium, the IP6 adsorption capacity of HApHS powder was approximately twice that of HAp-100 powder, indicating improved adsorption capacity. Consequently, the shaking time needed for IP6 adsorption on HApHS powder was determined to be 5 h.

### 3.3. Adsorption Isotherm of IP6

**Figure 6:**
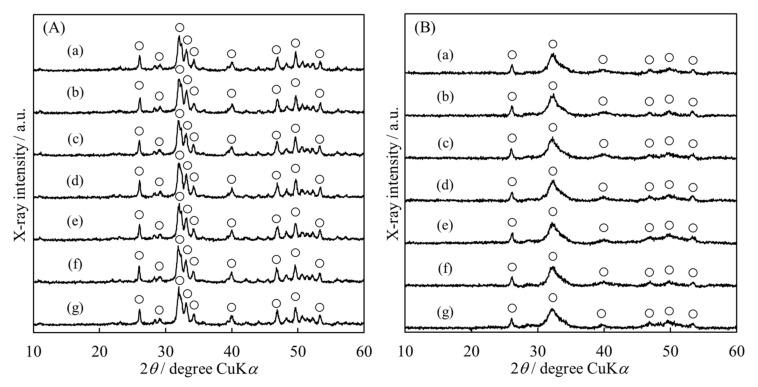
X-ray diffraction patterns of (**A**) HAp and (**B**) HApHS powder after adsorbing IP6. Initial concentrations of IP6 solution (mg/cm^3^): (a) 0, (b) 100, (c) 500, (d) 1000, (e) 2500, (f) 5000, (g) 10,000. 〇: HAp.

Figure 6 shows the XRD patterns of the HAp powder surface modified with various IP6 concentrations. We observed negligible change in any of the diffraction peaks and no dependence on the initial IP6 concentration of these peaks, indicating that the surface modification process had no effect on promoting the crystalline phase change. Therefore, we concluded that IP6 modified the surface of each HAp.

**Figure 7:**
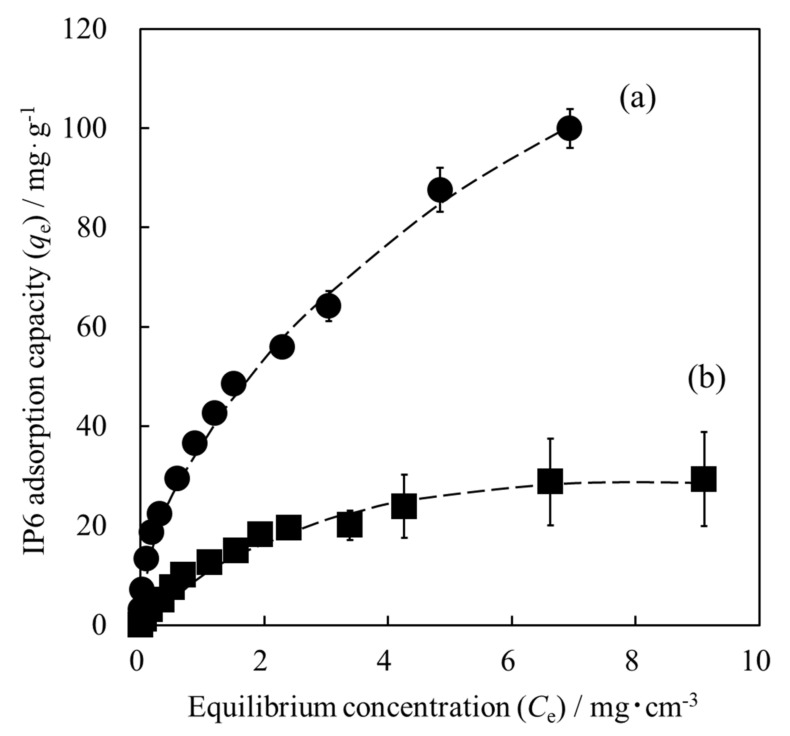
Adsorption isotherms of IP6 for different HAp surface (error bars: ±S.D., *n* = 3): (**a**) HapHS powder (●) and (**b**) HAp powder (■).

Figure 7 shows the adsorption isotherms of IP6 on each HAp powder. In the case of the HAp powder, the IP6 adsorption capacity gradually reached a plateau value as the initial IP6 concentration increased. Additionally, when the HApHS powder was used, the IP6 adsorption capacity was much higher than that of the HAp powder and continued to increase with the IP6 concentration. Despite not being shown here, when the unit of IP6 adsorption capacity was changed from [mg/g] to [mg/m^2^] in Figure 7, the adsorption isotherms of HAp and HApHS powders showed almost the same behavior. These results confirm that the IP6 adsorption capacity depends on the SSA. The two samples possess identical composition, except that the HapHS powder is a finer powder and has a higher SSA than the HAp powder. Therefore, we expect HapHS nanoparticles with high specific surface area to have high adsorption capacity for IP6. In addition to the above, we have also observed a negative shift in the surface potential of HAp adsorbed with IP6 [25].

We focused then on the adsorption mechanism of the IP6 on the HapHS powder. The experimental data on the adsorption of IP6 shown in Figure 7 were tested using the Langmuir and Freundlich isotherm models, as follows:

(4) $$\frac{C_{e}}{q_{e}}=\frac{1}{q_{max}K_{L}}+\frac{C_{e}}{q_{max}}$$

(5) $$\log q_{e}=\log K_{F}+\frac{1}{n}\log C_{e}$$

where *K_L_* is the Langmuir adsorption equilibrium constant, *q_max_* (mg/g) is the maximum IP6 adsorption capacity, *q_e_* (mg/g) is the amount of IP6 adsorption at equilibrium concentration, *C_e_* (mg/cm^3^) is the equilibrium concentration of IP6, and *K_F_* and *n* are the Freundlich adsorption equilibrium constants related to the adsorption capacity and intensity, respectively [32,33,34].

Table 2 summarizes the fitting of the experimental data to the Langmuir and Freundlich isotherm models. When the adsorption isotherm of IP6 on the surface of the HAp powder was fitted using the Langmuir model and the Freundlich model, the determination coefficient (*R*^2^) values were 0.9943 and 0.9581, respectively, indicating that the Langmuir model was the best fit. These results suggest that IP6 was adsorbed in a monolayer on the adsorption sites of the HAp powder surface [32,35]. Therefore, the adsorption was of chemical nature, chelate bond between IP6 and HAp. In general, it is common for the HAp as an adsorbent to fit the Langmuir model [36,37].

However, when the adsorption isotherm of IP6 on the surface of HApHS powder was fitted, the determination coefficient (*R*^2^) for the Langmuir model and the Freundlich model were 0.9163 and 0.9978, respectively. The Freundlich model was therefore more suitable to describe the adsorption of HApHS. These results suggest that the adsorption site on the HApHS powder surface is a heterogeneous surface and that IP6 is adsorbed in a multilayer [32,38]. The adsorption was both of chemical and physical nature, which is related to the nature of the nanoparticles and the high SSA of the HApHS powder.

## 4. Conclusions

HApHS powders with high SSAs were synthesized to improve the compressive strength of chelate-setting CPCs, and the adsorption properties of the HApHS powder on IP6 were examined. Ultrasonic irradiation during the wet synthesis process is crucial to obtain fine HAp particles with a high SSA. The HApHS powder was synthesized according to different ultrasonic irradiation time. The SSA of the HApHS(1) powder synthesized by ultrasonic irradiation was 214 m^2^/g, while that of the HApHS(0) powder synthesized without ultrasonic irradiation was 147 m^2^/g. Therefore, ultrasonic irradiation promoted the miniaturization of the particle size. Moreover, the IP6 adsorption capacity of the HApHS powder was larger than that of the commercial HAp powder because of the increased SSA. Regarding the adsorption mechanism of IP6 on the two types of HAp powder, the adsorption mechanism of the commercial HAp powder could be explained by the Langmuir model, whereas that on the HApHS powder followed the Freundlich model. The results show that IP6 is chemisorbed on commercial HAp powder, while IP6 is absorbed on HApHS powder by both physical and chemical adsorption. The HApHS powder presented here expected to be a novel starting material powder to be used to improve compressive strength of chelate-setting CPCs.

## Figures and Tables

**Table 1:** Specific surface area and Ca/P atomic ratio values of the powders synthesized after ultrasonic irradiation (error bars: ±S.D., *n* = 3).

HApHS(X) X/min	Specific Surface Area /m^2^/g	Ca/P Molar Ratio /-
0	147 ± 18	1.63 ± 0.02
1	214 ± 7	1.71 ± 0.03
2	211 ± 13	1.70 ± 0.03
3	223 ± 17	1.71 ± 0.01
4	204 ± 9	1.69 ± 0.04
5	216 ± 13	1.73 ± 0.08

**Table 2:** Fitting of IP6 adsorption isotherms to the Langmuir and Freundlich isotherm models.

Sample	Specific Surface Area /m^2^/g	Langmuir Model		Freundlich Model		
		*K_L_*	*R* ^2^	*K_F_*	*n*	*R* ^2^
HApHS	208	0.8173	0.9163	39.02	2.039	0.9978
HAp	62.9	0.5809	0.9943	9.977	1.572	0.9581

## Data Availability

The data presented in this study are available on request from the corresponding author.
